# From ancient Egypt to the dermatologic office: An overview of skin substitutes and modern‐day applications in dermatologic surgery

**DOI:** 10.1002/hsr2.1067

**Published:** 2023-01-16

**Authors:** Alison Tran, Sahira Desai, Deanne Mraz Robinson

**Affiliations:** ^1^ Menter Dermatology Research Institute Baylor University Medical Center Dallas Texas USA; ^2^ Heights Dermatology Houston Texas USA; ^3^ Modern Dermatology Westport Connecticut USA; ^4^ Yale School of Medicine Department of Dermatology New Haven Connecticut USA

**Keywords:** amniotic allograft, biologic, dermatologic surgery, skin substitutes (SS), synthetic

## Abstract

Skin grafting (specifically xenografting) dates back to as early as 1500 before Christ (BC) in the Ebers papyrus, an Egyptian medical papyrus. In 1503, the use of human skin allograft was described in the manuscript of Branca of Sicily, and among the Hindu Tilemaker Caste approximately 2500–3000 years ago, surgeons repaired defects secondary to nose amputations of those who committed adultery and thievery. Over the years, many advancements in skin grafts/substitutes and their applications have propelled the field to focus on better graft survival, contracture prevention, cosmesis, and quality of life. We provide a general overview of skin substitutes (SS) with a particular focus on placental SS and their current applications in dermatologic surgery.

## 1 INTRODUCTION

In the early 19th century, a few trials were reported: successful autografting of full‐thickness skin graft on a sheep (Boronio) and successful nose reconstruction (Bunger), but it was not until 1869 when Reverdin galvanized worldwide interest with the successful account of pinch grafting.^1^ In 1872, Ollier discovered the importance of the dermis in skin grafting, and by 1886, Thiersch discovered the importance of split‐thickness grafts in the coverage of large wounds.^2^ By 1893, full‐thickness skin grafting (FTSG) became popularized by Krause (also reported by Wolfe in 1875).^2^ In 1942, FTSGs were successfully used by Brown and McDowell to treat burns and in 1964, Tanner developed the Tanner‐Vandeput mesh dermatome, which was a device used to expand the surface area of split‐thickness skin grafts up to a ratio of 1:3.^2^ In 1975, Rheinwald and Green published the formation of epidermis like tissue through in vitro cultivation of epidermal keratinocytes, and in 1980, Connor and colleagues reported the first use of human cultured epidermal autografts (CEAs).^2^

## 2 WHAT ARE SS?

SS are a group of biologic, synthetic, or biosynthetic materials that enable temporary or permanent wound closure by replacing the functions of the skin. When placed on the injury, they prevent bacterial colonization and trauma to the wound. The elements of the SS also accelerate repair by generating a beneficial healing environment. Over the years, there have been many proposed ways to define SS and its subcomponents. Table 1 illustrates Kumar's three‐category system^3^: (1) temporary impervious dressing materials, (2) single‐layer durable SS, and (3) composite SS,^1^, ^4^ which include skin grafts such as xenografts and allografts. Xenografts are derived from animals, with porcine and bovine being the most commonly used. They are successful in treating exposed bone, tendons, and cartilage by creating dermal regeneration and restoration while still maintaining the integrity and mobility of the existing area. Allografts are sourced from humans and can be further divided into dermal, epidermal, and composite allografts.

**Table 1:** Kumar's three category system

Class I: Temporary impervious dressing materials
1. Single‐layered 1.1. Biological dressing substitute, for example, amniotic membrane or potato peel 1.2. Synthetic dressing substitute, for example, synthetic polymer sheet, polymer foam/spray 2. Bi‐layered 2.1. Tissue engineered material, for example, polymer membrane and fibroblasts cells on nylon mesh
Class II: Single‐layer durable skin substitutes
1. Epidermal substitutes, for example, cultured epithelial autograft (CEA) 2. Dermal substitutes, for example, bovine collagen sheet, porcine collagen sheet, bovine, or human dermal matrix
Class III: Composite skin substitutes
1. Skin grafts, for example, xenografts and allografts 2. Tissue engineered skin, for example, Integra®, Biobrane®

Alternatively, SS can be classified based on durability (temporary/permanent) (Table 2) and type (synthetic/biological). Recently, there has been a push toward establishing a universal classification system inspired by factorial design, whereby an algorithm is used to search for a SS with consideration of multiple factors: (1) cellularity (acellular or cellular), (2) layering (single layer or bilayer), (3) targeted area of skin to be replaced (dermal, epidermal or both), (4) materials used (synthetic, biological or both), and (5) permanence (temporary or permanent).^4^

**Table 2:** Classification based on durability and its indications

Durability	Indications
Temporary skin substitutes	Temporary physical barrier, wound closure, mechanical trauma protection, fosters moist wound environment
Permanent skin substitutes	Permanent wound closure—particularly for deep dermal or full thickness burns, higher quality skin replacement

### 2.1 Synthetic SS

Synthetic SS (SSS) are comprised of stable biodegradable immunocompatible polymers that provide three‐dimensional structural support and an environment conducive to tissue regeneration. Ideally, they should last for at least 3 weeks to allow for neovascularization, fibroblast, and epithelial cells formation followed by natural biodegradation.^2^ SSS have an advantage of precision/control and enhanced effect depending on additive growth factors and matrix components with the additional benefit of reduced risk for disease transmission.^2^ However, they may not be as useful when trying to produce a biologically compatible material as they typically lack basement membranes, and thus do not resemble native skin. Table 3 outlines some of the currently available SSS in the market.

**Table 3:** Current synthetic substitutes, composition, and indications

Synthetic skin substitute	Composition	Indications
Biobrane® (UDL Laboratories, Inc., Rockford, IL, USA)	Acellular, bi‐layered: inner nylon or silicone mesh layer enables fibrovascular ingrowth and outer silastic layer (porcine collagen) serves as a bacterial barrier	Can replace dermis and epidermis (full‐thickness substitute), and is used in the management of burns, particularly partial‐thickness until wound healing is achieved
Dermagraft® (Intercytex Ltd., Manchester, UK)	Cellular (allogenic neonatal fibroblasts), single‐layered: synthetic bioabsorbable polyglactin mesh impregnated with allogenic neonatal fibroblasts	Dermal substitute used for burns, chronic wounds, and ulcers
Integra® (Integra LifeSciences Corp., Plainsboro, NJ, USA)	Acellular, bi‐layered: silastic silicone membrane, bovine collagen Type I dermal analog that integrates with the patient's cells and incites dermal regeneration	Can replace dermis and epidermis (full‐thickness substitute, FTS), and is used for deep partial‐thickness and full‐thickness burn wounds, full‐thickness skin defects, chronic wounds and soft tissue defects
Apligraft® (Organogenesis, Inc., Canton, MA, USA, and Novartis Pharmaceuticals Corp., East Hanover, NJ, USA)	Cellular (allogenic neonatal keratinocytes and fibroblasts), bi‐layered: Type I bovine collagen and allogenic keratinocytes and neonatal fibroblasts	Can replace dermis and epidermis (FTS), and is used for partial to full‐thickness burns, skin graft donor sites, chronic wounds, diabetic ulcers, and epidermolysis bullosa
Matriderm® (MedSkin Solutions Dr Suwelack AG, Billerbeck, Germany)	Acellular, single‐layered: bovine Type 1 collagen matrix provides structural support for ingrowth of blood vessels and cells, and α‐elastin hydrolysate improves stability and elasticity of regenerating tissue Fibroblasts lay down extracellular matrix and the Matriderm resorbs as the healing process continues	Dermal substitute used for full‐thickness or deep dermal burns, and chronic wounds
OrCel® (Ortec International, Inc., New York, NY, USA)	Cellular (allogenic neonatal keratinocytes and fibroblasts), bi‐layered: cellular matrix with normal human allogenic keratinocytes and dermal fibroblasts cultured into two separate layers into a Type I bovine collagen sponge	Can replace dermis and epidermis (FTS) and is used for chronic wounds and skin graft donor sites
Hyalomatrix® (Anika Therapeutics, Inc., Bedford, MA, USA)	Acellular, bi‐layered: hyaluronan base scaffold (delivers hyaluronan to wound bed) with autologous fibroblast and outer silicone membrane (temporary epidermal barrier)	Can replace dermis and epidermis (FTS) and is used for burn wounds and chronic wounds

*Note*: Not an exhaustive list.

Adapted from: Halim et al.^1^ and Davison‐Kotler et al.^4^

### 2.2 Biological SS

Biological SS (BSS) have a more intact extracellular matrix (ECM) structure and a basement membrane, which allows for a more natural dermis and re‐epithelialization. They have the advantage of being relatively less expensive and more abundant in supply than synthetic substitutes. However, due to its natural components, there may be issues with revascularization in comparison to synthetic substitutes. The most common BSS used are porcine skin xenograft, skin allograft, and amnion^2^ (Table 4). Xenografts are used to temporarily cover wounds and will not revascularize. Allografts are cadaveric skin grafts that are used to prepare a wound bed for autografting and have an initial revascularization period. Autografts are taken from the patient and have the advantage of permanent skin healing through antigenic compatibility. CEAs are prepared from isolated keratinocytes from a full‐thickness skin biopsy and then incorporated and expanded into a neoepidermis. Dermal substitutes are obtained from cadaveric allografts and are comprised of a matrix of glycosaminoglycans and collagen.^5^ More recently, multilayered substitutes have garnered some attention. They are typically prepared from a dermal–epidermal junction biopsy, in which a suspension composed of autologous keratinocytes, fibroblasts, and melanocytes is sprayed onto a wound. The silicone sheet, which is composed of collagen and glycosaminoglycans, serves as an epidermis that encourages neovascularization for 2–3 weeks while the allograft matrix degrades and is replaced by the host's collagen matrix.^5^

**Table 4:** Current biological substitutes, composition, and indications

Biological substitute	Composition	Indication
Xenograft	Harvested from animals	Temporary graft for clean partial‐thickness burns
Skin allograft	Harvested from cadavers (cryopreserved and glycerol‐preserved) or living donors	Wound bed preparation, definitive dressing, sandwich grafting technique, interim coverage after burn scar release
Cultured epithelial autograft (CEA)	Cultured keratinocytes and expanded into sheets	Extensive full‐thickness burns
Amnion/placental	Thin tissue from innermost layer of fetal membrane harvested from placenta of screened donors	Partial‐thickness burns, facial burns, temporary coverage in wound bed preparation and sandwich grafting technique

Modified from: Halim et al.^1^

## 3 GENERAL APPLICATION

SSS and BSS can be used for a variety of surgical procedures. Generally, SS have been used to re‐establish the skin barrier in reconstructive surgery for burns, traumas (postextensive excision), non‐healing (including diabetic foot wounds), or large wounds that will not close via primary or secondary intention or as supportive material for various repairs, for example, hernia, abdominal wall, and reconstructions, for example, abdominal, breast, gynecological.^6^

The effectiveness of a specific scaffold is not only contingent upon a surgical technique that enables injuries to heal in an accelerated, non‐destructive manner with minimal risks of skin injury, but also its specific characteristics. The efficacy of a specific SS may differ depending on host tolerability, propensity for rapid vascularization, and the anatomic region for which it is applied. BSS may be preferred over synthetic materials in some cases such as abdominal wall repair as synthetic materials have been associated with higher incidences of seroma/hematoma, infection, and pain as well as fistula formation and skin erosion.^6^ Further, tolerability is especially important in abdominal procedures due to the risk for adhesions; thus, an ideal graft would be well‐tolerated, and support vascularization and infiltration of host cells with minimal scarring.^6^ Cellular infiltration observed with the use of dermal biologic SS is effective for wound closure while minimizing wound contraction and consequential hypertrophic scarring.^6^ Regardless of clinical application, SS ideally serve to protect the patient from increased risk of infections, complications, insensible water loss as well as facilitate all phases of wound healing: inflammatory with fibroblast infiltration, proliferative, maturation, or remodeling phase while also avoiding host rejection. Second, patient‐related outcomes such as pain, cosmesis, and ultimately, quality of life need to be taken into full consideration when choosing between SS. There has been a growing interest in the use of placental SS in postoperative care following Mohs micrographic surgery (MMS) as it is immunologically privileged^7^; thus there is no risk of rejection when used in transplant with grafts or flaps.

## 4 PLACENTAL SS IN DERMATOLOGY

The placenta is a specialized organ that serves as a materno‐fetal interface enabling the transfer of nutrients, gases, hormones, and cytokines, including insulin‐like growth factor‐1, epidermal growth factor, platelet‐derived growth factor, fibroblast growth factor‐2, vascular endothelial growth factor, and transforming growth factor‐β.^8^ The placenta is composed of three layers; the innermost amniotic layer surrounds the embryo/fetus and consists of a single‐celled epithelial layer firmly fixed to a deeper collagen containing mesodermal layer, the middle allantois layer is rich in blood supply, and the outermost chorion which comes into contact with the endometrium and consists of trophoblastic and mesenchymal tissue.^9^ Placental tissue effectively supports wound healing through a rich ECM‐containing proteins (collagens I, III, IV, VI, proteoglycans, glycoproteins), growth factors, cytokines, and viable endogenous cells and mesenchymal stem cells that help facilitate the wound‐healing process.^10^, ^11^ Further, these various components impact cell differentiation, hormone/protein production, and basement membrane remodeling.^10^ Such properties are beneficial for the treatment of complex, chronic, nonhealing burns, ulcers, and wounds.

The amnion is the dominant membrane used in placental skin substitutes (PSS) due to ease of separation and purification compared to the chorion.^12^ Further, the amnion has antibacterial, anti‐inflammatory, and antiscarring properties.^13^ Its stromal layer contains neonatal fibroblasts and mesenchymal stem cells. Though the chorion also contains a stromal layer, its trophoblast layer contains high levels of inflammatory cytokines and proteases (at term) and facilitates extracellular placental matrix degradation. Human amniotic membrane (HAM) allografts can either be single or multilayered. Monolayer HAM consists of only amnion while bilayer HAM consists of amnion and chorion. The newer tri‐layered allograft membrane consists of a chorion layer sandwiched between two layers of amnion,^11^ which has the added benefit of improved mechanical handling properties over its single and bi‐layered counterparts and stimulates pro‐healing cellular responses while dampening pro‐inflammatory responses.^11^

The use of placental tissue as an allograft during a skin transplantation was first documented in 1910.^9^ It has been used extensively in chronic wounds, including that of the lower extremities, necrolysis secondary to burns and in bullous disease, for example, all studied patients with Stevens–Johnson syndrome and toxic epidermal necrolysis demonstrated accelerated wound repair/increased survival, and great cosmesis,^10^ and post MMS as it has many advantages over standard wound care.

## 5 PREPARATION

The standard procedure for preparing placental transplantation begins with obtaining the material from the donor, which is often from donation to the Tissue Bank at the time of birth. This usually occurs during cesarean sections as it allows aseptic procurements devoid of passage through the birth canal.^10^ Donors are then screened for infectious diseases including human immunodeficiency virus (HIV), hepatitis B and C, and syphilis.

The first stage of preparation begins with washing the placenta in normal saline and placing the placenta into a solution of normal saline and antibiotics, which may vary across hospitals. The tissue is stored in a special refrigerator at +4°C. The second stage consists of the purification process whereby the placenta undergoes five sterile rinsing cycles in normal saline, on a shaker at +4°C until clean solution is obtained. The amnion is then separated from the chorion and marked to preserve its anatomical position/orientation as the epithelial side will lay directly on a carrier and then will be applied with the basement membrane directly outward post‐wound application as this will stimulate epithelization via migration, adhesion, and cell proliferation.^10^ The transplant can be optionally sterilized with gamma or electron beam radiation to further decrease risks for infection. Transplants are properly stored via cryopreservation (−80 or −150°C) with cryoprotectants or dehydration to prevent tissue damage by reducing chemical and enzymatic activity and inhibiting growth of microorganisms.^10^ Additional variants in the preparation of placental grafts include perforation and cross‐linking, which are beyond the scope of this paper.

## 6 UTILITY IN DERMATOLOGIC SURGERY

The incidences of both melanoma and non‐melanoma skin cancers (NMSC) are increasing worldwide. The current standard of treatment for NMSC is MMS which is a tissue‐sparing surgical modality for skin cancer removal developed by Dr. Frederick Mohs in 1941.^14^ MMS has high cure rates (1% 5‐year recurrence for BCC and less than 6% 5‐year recurrence for SCC)^15^, ^16^, ^17^ and is recommended for higher‐risk BCC and SCC lesions, which are greater than 2 cm in size, in immunocompromised patients, for example, organ transplant recipients, recurrent/incompletely excised, aggressive and/or with perineural invasion, with positive margins, and ulcerated, with burns or previously irradiated.^17^ Additionally, it is often recommended for treating various anatomical regions where tissue conservation is imperative, for example, ears, eyes, nose, and lips.^18^

MMS typically involves the removal of approximately 0–2 mm margin of normal tissue along with the visible tumor, followed by a 30–40‐min fixation process before the tissue is cut into horizontal frozen sections and examined under the microscope. Tissue positive for tumor is marked, and additional tissue is sampled from the marked tissue in stages until clear tissue margins are obtained, generally in two cutting stages.^14^ Though MMS is a tissue‐conserving surgical modality, repeated surgical intervention may be required to achieve adequate removal of abnormal tissue. Further, larger and deeper wounds may require reconstruction with skin flaps or substitutes.

Several recent studies have demonstrated the advantages of using PSS in MMS‐related wounds compared with the current standard of care procedures.^19^ Among the advantages include ease of postprocedural wound care as patients can get weekly dressing changes versus traditional healing by secondary intent which requires daily dressing changes^19^ as well as lower risk of infection. Surgical site infection rates for below‐the‐knee surgeries with MMS and wide local excisions range from 2.3% to 8.3%.^18^ Further, since it involves treating anatomically sensitive areas, cosmesis is often a concern, and PSS post‐MMS has been associated with better cosmesis/less need for scar revision.^19^ This is especially important for patients who are poorer surgical candidates or are prone to scarring as they may have a higher risk for postsurgical sequelae and complications, for example, infection, bleeding, damage to nearby structures, wound dehiscence, pain, and scarring (particularly in scar/keloid prone individuals).^16^, ^18^

## 7 CONCLUSION

The use of SS for soft tissue repair/defect can positively impact the quality of life for patients. Its application can have a life‐altering impact on patients who are unable to heal, have extensive injuries/burns, or have insufficient skin available for autografting or flaps. Some of SS functions are to re‐establish the skin barrier, reduce insensible fluid loss and act as a covering to prevent bacterial colonization.

Even with the positive results that SS have shown, there are still some shortcomings that must be considered. Synthesized materials cannot fully and accurately replace the inherent roles that the skin carries out. In regard to visual appearances, no SS has yet replicated what uninjured skin looks like. Arranging the wound bed is also an obstacle when dealing with SS that need revascularization because there is a possibility of infection and risk for rejection. The risk of graft failure may be secondary to fluid accumulation, infection, shearing, excessive tension, or poor wound bed vascularity. In addition, postprocedural sequelae such as hypopigmentation, hyperpigmentation, scarring, and infection transmission can occur, leading to an inadequate esthetic appearance. Though complications may be mitigated with proper wound bed preparation and hemostasis and graft protection with secure dressings, they cannot be entirely eradicated.

Placental tissues include the placental disc, umbilical cord, amniotic fluid, and amniotic sac which is further divided into the amnion and chorion. Placental substitutes have been used since the early 20th century and have been demonstrated to have significant benefits in the treatment of burns, chronic wounds/diabetic foot ulcers, and surgery across multiple specialties including, but not limited to, obstetrics, general, neurosurgery, dental, ophthalmologic, and dermatology. Since placental tissues carry multipotent mesenchymal cells, it is immunologically privileged so there is no risk of rejection when used in transplants/grafts. Furthermore, placental tissue can support rapid tissue repair and affects all phases of wound healing and tissue remodeling. Additionally, there is potential for achieving great cosmesis, which is not only determined by timely wound debridement and treatment but also due to no risk of PSS rejection and its unique antifibrotic and anti‐inflammatory properties. The major limitations of placental tissue use include costly treatments, ethical concerns regarding repurposing human tissue, patient preference and lack of clinical trials and randomized control studies with sufficient data demonstrating its effectiveness.

Although there has not yet been a “perfect” SS developed, the current array of available SS has greatly broadened the arena for dermatologists aiming to heal substantial wounds. Having a background knowledge of what they are, how they were derived, their applications in medicine, and their flaws can allow for the most effective and productive use of SS in dermatology.

## AUTHOR CONTRIBUTIONS

**Alison Tran**: Conceptualization; supervision; writing – original draft; writing – review & editing. **Sahira Desai**: Conceptualization; writing – original draft. **Deanne Mraz Robinson**: Conceptualization; supervision; writing – review & editing.

## TRANSPARENCY STATEMENT

The lead author Alison Tran affirms that this manuscript is an honest, accurate, and transparent account of the study being reported; that no important aspects of the study have been omitted; and that any discrepancies from the study as planned (and, if relevant, registered) have been explained.

## Data Availability

Data sharing is not applicable to this article.
